# Phosphodiesterase 1b (PDE1B) Regulates Spatial and Contextual Memory in Hippocampus

**DOI:** 10.3389/fnmol.2019.00021

**Published:** 2019-02-07

**Authors:** Susan McQuown, Shouzhen Xia, Karsten Baumgärtel, Richard Barido, Gary Anderson, Brian Dyck, Roderick Scott, Marco Peters

**Affiliations:** Dart NeuroScience, LLC, San Diego, CA, United States

**Keywords:** PDE1, PDE1B, phosphodiesterase (PDE), memory consolidation, spatial memory, Barnes Maze, hippocampus, CIAS

## Abstract

Augmentation of cyclic nucleotide signaling through inhibition of phosphodiesterase (PDE) activity has long been understood to enhance memory. Efforts in this domain have focused predominantly on PDE4, a cAMP-specific phosphodiesterase implicated in consolidation. But less is known about the function of other PDEs expressed in neuroanatomical regions critical to memory. The PDE1 isoforms are the only PDEs to regulate neuronal cAMP and cGMP levels in a Ca^2+^/Calmodulin (CaM) dependent manner. Here, we show that knock-down of PDE1B in hippocampus of adult mice enhances contextual and spatial memory without effect on non-cognitive behaviors. Pharmacological augmentation of memory in rats was observed with a selective inhibitor of PDE1 dosed before and immediately after training, but not with drug dosed either 1 h after training or before recall. Our data clearly demonstrate a role for the PDE1B isoforms as negative regulators of memory, and they implicate PDE1 in an early phase of consolidation, but not retrieval. Inhibition of PDE1B is a promising therapeutic mechanism for treating memory impairment.

## Introduction

There is great need for therapies for cognitive deficits in patients suffering from schizophrenia, depression, and neurodegenerative conditions including Alzheimer’s and Parkinson’s disease. Considerable efforts have focused on targets implicated in dopaminergic (DA) signaling, because of the importance of prefrontal D1 receptors (D1-R) in attention and working memory (Arnsten et al., 1994, 2017; Goldman-Rakic et al., 2004), and of hippocampal D1-R in memory consolidation (O’Carroll et al., 2006; Rossato et al., 2009; Bethus et al., 2010). The pro-cognitive effects of D1-R are mediated via g-protein regulation of cyclic adenosine-monophosphate (cAMP) signaling, leading to activation of protein kinase A (PKA) to modulate synaptic plasticity and memory. The actions of cAMP are counteracted by phosphodiesterase (PDE) activity that rapidly hydrolyzes the second messenger and terminates the receptor signal. Inhibitors of the cAMP-specific PDE4 enhance memory and DA signaling (Kuroiwa et al., 2012), but the target is associated with dose limiting complications including emesis (du Sert et al., 2012). More recently, medicinal chemistry efforts have led to the discovery of selective inhibitors of other PDEs, including the dual substrate specific phosphodiesterases PDE1 and PDE2 (Schmidt, 2010; Maurice et al., 2014). First isolated almost 50 years ago from bovine and rat brain (Cheung, 1970; Kakiuchi and Yamazaki, 1970), the type 1 phosphodiesterases (PDE1) are characterized by Ca^2+^-dependent stimulation via the Ca^2+^-binding protein calmodulin (CaM) and are as such the only PDE to regulate neuronal cAMP and cGMP levels in a Ca^2+^-dependent manner (Goraya and Cooper, 2005). Three isoforms of CaM-PDEs are expressed in CNS: PDE1A – located most prominently to the CA1–4 area of the hippocampal formation and in cerebral cortex, PDE1B – expressed prominently in regions with strong dopaminergic innervation such as dentate gyrus, striatum, and in prefrontal cortex, PDE1C – located to cerebellum and olfactory epithelium (Polli and Kincaid, 1992, 1994; Yan et al., 1994, 1996). The function of these isoforms is poorly understood. Knockout of PDE1B in mice increases D1R mediated PKA activation, phosphorylation of striatal DARPP-32 (dopamine and cAMP regulated phosphoprotein), and it causes exaggerated locomotor responses to methamphetamine administration (Reed et al., 2002; Ehrman et al., 2006; Siuciak et al., 2007). This phenotype is overall consistent with coupling of PDE1B to D1R within the direct pathway in striatum (Nishi and Snyder, 2010). Initial studies on PDE1B knockout mice reported spatial learning deficits in the Morris Water Maze (Reed et al., 2002), but these findings were not corroborated in subsequent studies (Ehrman et al., 2006; Siuciak et al., 2007) – leaving the role of PDE1B in memory unclear.

Here, we address the function of PDE1B in spatial and contextual memory. We show that RNA interference knockdown of PDE1B in hippocampus enhances memory, and we provide pharmacological evidence for a specific role for PDE1 in memory consolidation.

## Materials and Methods

### Experimental Subjects

All animal work adhered to protocols approved by the IACUC committee of Dart NeuroScience, LLC and followed the guidance of the National Research Council Guide for the Care and Use of Laboratory Animals Studies (2011). We used male C57Bl/6 × 129SvTac hybrid mice (Taconic Farms) for *Pde1b* knockdown and memory studies, and male Long-Evans rats (Envigo, United States) for pharmacology. Mice were housed in groups of four, and rats in groups of two, maintained on a 12 h light/dark schedule, and allowed *ad libitum* access to food and water. Experiments were conducted on 3–6 month old male mice during the light phase. Rats were 360–430 g (approximately 3 month old) at the initiation of study.

### Contextual Fear Conditioning Task (cFC)

Fear conditioning was conducted using conditioning chambers fitted with a stainless-steel grid floor through which footshocks can be delivered (mice: Coulbourn Instruments, PA, United States; rats: Med Associates Inc., VT, United States). Protocols were developed to demonstrate the effect of intra-hippocampal manipulations, including post-trial inhibition of PDE4 by Rolipram (Li et al., 2011) and blocking NMDA receptors prior to training (data not shown). Training consisted of placing an animal in the chamber and after 120 s delivering two (to induce a weak memory) electrical footshocks (2 s duration; 0.4 mA) separated by a 60 s inter-trial interval (ITI). Experimental subjects were returned to the home cage 30 s after the final footshock. The percentage of time spent freezing during 3 min of re-exposure to the training context as a measure of memory was recorded automatically using Video Freeze software. Based on prior experiments, sample size was set to detect a 22% difference in freezing in mouse cFC (shRNA) with power = 0.8 in mouse cFC (shRNA), and a 25% difference in freezing in rat cFC (pharmacology) with power = 0.9.

### Open Field Exploration

Mice were allowed to explore square open field chambers (40 cm W × 40 cm D × 35 cm H) filled with cobb bedding under dim light (60 lux) for 10 min each day on two consecutive days. Mice were placed in the arena and motion was recorded automatically using EthoVision 8.5 tracking software (Noldus Information Technology, Netherlands). We calculated the distance moved across the arena as a measure of horizontal activity, and the time spent in the center or perimeter of the arena as a measure of anxiety related behavior.

### Spatial Memory in the Barnes Maze

All studies were carried out on a circular platform (36” in diameter) with 20 holes (2” in diameter) around the perimeter (San Diego Instruments, United States). A removable escape box was placed beneath the target hole. Overhead lights provided motivation for the animal to seek the escape box. Several large, salient objects were placed around the maze to provide proximal visual cues. A camera was suspended from the ceiling above the platform to permit automated tracking of experimental subjects. Before the first training trial, mice were familiarized to escape the maze by placing the subject directly in front of the target position and guiding the animal into the escape box in a no-cue environment. At the start of each training trial, mice were placed in the center of the platform inside an opaque start tube for 15 s and then released. The trial ended when the subject entered the escape box. If at the end of 300 s the subject had not found the escape box, the mouse was guided to it and a latency of 300 s was scored. Latency to escape and errors were scored as measures of acquisition. Mice remained in the escape box 30 s before returning to the home cage. The maze and escape box were cleaned with 70% ethanol solution to dissipate odor cues and provide a standard olfactory context for each trial. Escape holes were counterbalanced across the experiment. Training was conducted twice a day with an ITI of one min for two days. Twenty-four hour later a probe-test was conducted. The escape box was removed and the mouse’s exploration monitored for 120 s. Locomotion during the 120 s probe-trial, time spent in the correct quadrant (TQ), number of errors prior to the first correct escape visit, and search strategy were determined. All trials were tracked and recorded by EthoVision 8.5 (Noldus, Inc.).

### *In vivo* RNA Interference

All vectors were constructed at Vector Biolabs (Malvern, PA, United States). AAV2 ITR containing plasmids (Vector Biolabs) were packaged with AAV5 capsid. shRNA targeting *Pde1b* (TRCN0000115001, target sequence: GCCTCCAAGTTTCTAAGCAAT) was expressed from a U6 promoter. eGFP (to monitor *in vivo* transduction by AAV5) was co-expressed from a CAG promoter (a hybrid of the CMV early enhancer element and chicken beta-actin promoter, Vector Biolabs Cat. No: 7073) contained in the same vector. shRNA targeting eGFP (RHS4459, target sequence: TACAACAGCCACAACGTCTAT) was used to control for non-specific effects of viral transduction and shRNA expression on memory formation. The Efficiency to knock-down *Pde1b* mRNA was confirmed in cultured mouse hippocampal neurons *in vitro* using RT-PCR on a StepOnePlus Real-Time PCR System (Thermo Fisher Scientific). Data was normalized to *Gapdh* and ΔΔCT values were determined relative to control. For virus injection, mice were anesthetized with a Ketamine/Xylazine anesthetic (100 mg/kg, 10 mg/kg), core body temperature maintained throughout the surgery using a heat blanket, and an ophthalmic ointment applied. Bregma and lambda were leveled to the same plane, as were two points 2 mm of each side of the midline. Holes were drilled at stereotactic coordinates AP = –1.5 mm, Lateral = ±1.5 mm and the injection cannula was lowered 1.75 mm below the surface of the skull. One microliter of virus was injected bilaterally at a rate of 0.5 μl/min, and after 1 min the cannula was pulled up to –1.5 mm and another 1 μl was injected (5 × 10^10^ AAV particles). After an additional 1 min the cannula was removed, a thin layer of bone wax applied, the skin closed above the scalp, and post-surgery care provided. Mice were allowed to recover for 14–21 days prior to experimentation.

### Drug Treatment

6-(4-Methoxybenzyl)-9- ((tetrahydro-2H-pyran-4-yl)methyl)-8, 9,10,11-tetrahydro- pyrido[4’,3’:4,5]thieno[3,2-e][1,2,4]triazolo [1,5-c]pyrimidin- 5(6H)-one (compound 16-k, referred to as DNS-0056, ref. 25) was used to test the effects of pharmacological inhibition of PDE1 on contextual memory in rat. The drug was formulated in a vehicle of 10% DMSO, 30% PEG 400 and 60% saline, and administered orally at a dose of 0.3 mg/kg in a volume of 2 ml/kg. The same dose of DNS-0056 was previously shown to enhance object recognition memory in rats. Pharmacokinetic properties and PDE-selectivity of DNS-0056 are described in Dyck et al. (2017a).

### Immunohistochemistry

Anesthetized mice were transcardially perfused with PBS for 2 min followed by 4% PFA for 5 min, and brains post-fixed overnight in 4% PFA. Free-floating 50 μm coronal sections were prepared using a vibratome. Sections were blocked and permeabilized with 1% Triton X, 10% normal goat serum in PBS (pH7.4) for 1 h at room temperature and stained with anti-PDE1B rabbit polyclonal antibody (Cell signaling; 1:750) over night at 4°C. Sections were washed in PBS three times for 10 min and then stained with Alexafluor 546 goat anti-rabbit secondary antibody (Molecular probes; 1:1,000) using the same blocking solution for 3 h at room temperature. After a series of washes with PBS sections were mounted using a DAPI containing ProLong mounting agent (Invitrogen; 1:1,000). Slide mounted sections were imaged using a Zeiss LSM 780 confocal microscope using a 10× objective and Zen software (Zeiss, Jena, Germany). Image analysis was performed using the MetaMorph Microscopy Automation and Image Analysis software package (Molecular Devices, Sunnyvale, CA). Total signal intensity in the dentate gyrus was normalized to the superior half of the striatum for each image. At least six images were averaged for each individual mouse. Only images with GFP expression in the dentate gyrus were included in the analysis.

### Statistical Analysis

All experiments and analysis were performed blind to the treatment condition. Behavioral and biochemical data were analyzed by unpaired *t*-test (where appropriate) or by ANOVA, followed by *post-hoc* analysis to interrogate differences amongst individual treatment groups. Values greater than three standard deviations from the mean of each group were excluded as statistical outliers. Distribution of search patterns in the Barnes Maze were analyzed by Chi-squared test. Error bars represent s.e.m. in all figures.

## Results

**FIGURE 1:**
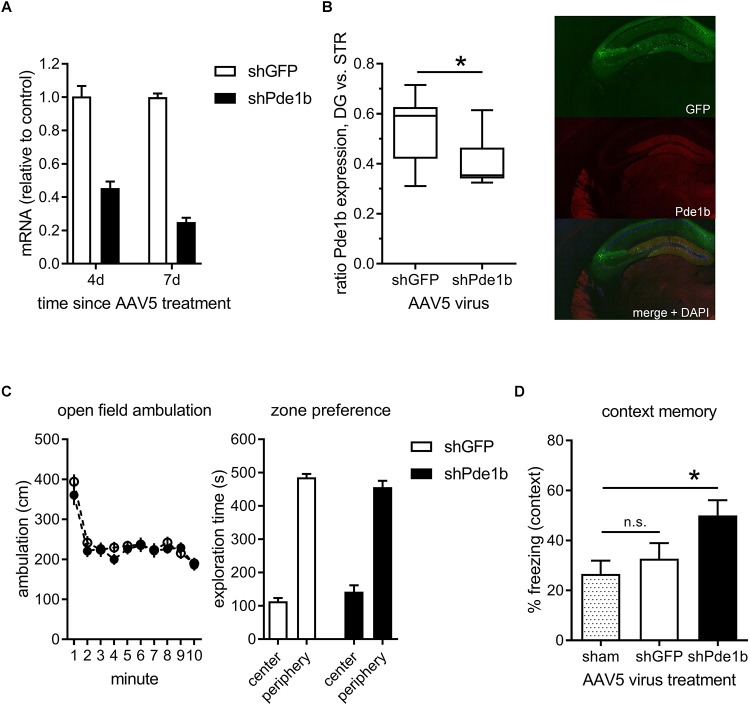
Effects of intra-hippocampal RNAi knock-down of PDE1B on open field exploration and contextual fear memory. **(A)** *Pde1b* mRNA levels measured in hippocampal cultures treated with shGFP control or shPde1b expressing AAV5 (*n* = 4 per treatment). PDE1B was efficiently targeted by the shRNA. **(B)** PDE1b protein expression 3 weeks after transfection with shPde1b or control AAV. Left: Box and whisker plots showing the expression of PDE1B in DG normalized to staining intensity in STR of the same animals (shGFP: *n* = 12; shPde1b: *n* = 15). A significant reduction in the relative expression of PDE1B protein between DG and STR was observed after shPde1b treatment. Right: Example image showing green fluorescence derived from a GFP marker co-expressed form the AAV5-shRNA vector, and PDE1B protein expression. **(C)** Open field exploration in mice treated with shPde1b (*n* = 20) or shGFP control (*n* = 20) virus. Left: Ambulation over the 10 min trial duration. Right: Time in the center and periphery of the open field. No difference was found between treatment groups. **(D)** Contextual fear memory was enhanced by PDE1B knockdown. Time spent freezing upon re-exposure to the training context is shown. shPde1b (*n* = 17), but not shGFP (*n* = 17), treated mice froze significantly more than sham controls (*n* = 13) on test. The mean ± s.e.m. is shown for all groups. Significant differences from vehicle control are indicated by an asterisk (^∗^).

To target PDE1B selectively we designed a short hairpin RNA, packaged it into AAV5, and tested it in cultures of hippocampal neurons (Figure 1A). When compared to shGFP control transfected neurons, *Pde1b* mRNA levels were reduced by 75% within 1 week of viral transduction (4d: 45.4%, 7d: 25.0% of control). We then stereotactically injected the AAV5 containing the shRNA constructs into the hippocampus of mice and tested knockdown of PDE1B protein approximately 3 weeks later (Figure 1B). GFP, co-expressed from AAV5, indicated widespread transduction of neurons in the hippocampal formation with prominent labeling of granule cells in DG *in vivo*. The ratio of PDE1B protein expressed in DG vs. striatum (STR) was significantly reduced in shPde1b treated mice when compared to shGFP controls (*t*(25) = 2.93, *p* < 0.01), indicating knockdown of PDE1B *in vivo*.

Constitutive (Reed et al., 2002; Siuciak et al., 2007) as well as conditional (Hufgard et al., 2017) PDE1B knockout mice exhibit altered exploratory activity in the open field. We therefore asked if selectively targeting PDE1B in hippocampus of adult mice causes locomotor effects that may confound analysis of a role in memory using the current approach. shPde1b treated mice and controls were allowed to explore an open field environment and activity monitored for 10 min (Figure 1C). There was no difference in ambulation as a measure of overall activity (effect of trial: *F*_(9,342)_ = 29.72, *p* < 0.0001; effect of gene and interaction: not significant; RM-ANOVA), or in the time spent exploring the center zone of the arena as a measure of putative anxiogenic effects. We thus continued to test the effects of hippocampal knockdown on contextual fear conditioning, memory of which is disrupted by hippocampal lesions (Phillips and LeDoux, 1992). Mice were trained with 2 CS-US pairings to induce a weak memory in controls, and tested 24 h later. A significant treatment effect on contextual fear was observed (one-way ANOVA: *F*_(2,44)_ = 4.07, *p* < 0.05). *Post hoc* analysis revealed that shPde1b treated mice froze significantly more than sham controls upon re-exposure to the training context (*p* < 0.05; Dunnett’s multiple comparison’s test), while no difference was found between shGFP and sham treated mice. We repeated the experiment to compare the AAV-shRNA treated groups only, and significantly more freezing was observed after shPde1b treatment (data not shown). Thus, hippocampal knockdown of PDE1B enhances contextual memory in mice.

**FIGURE 2:**
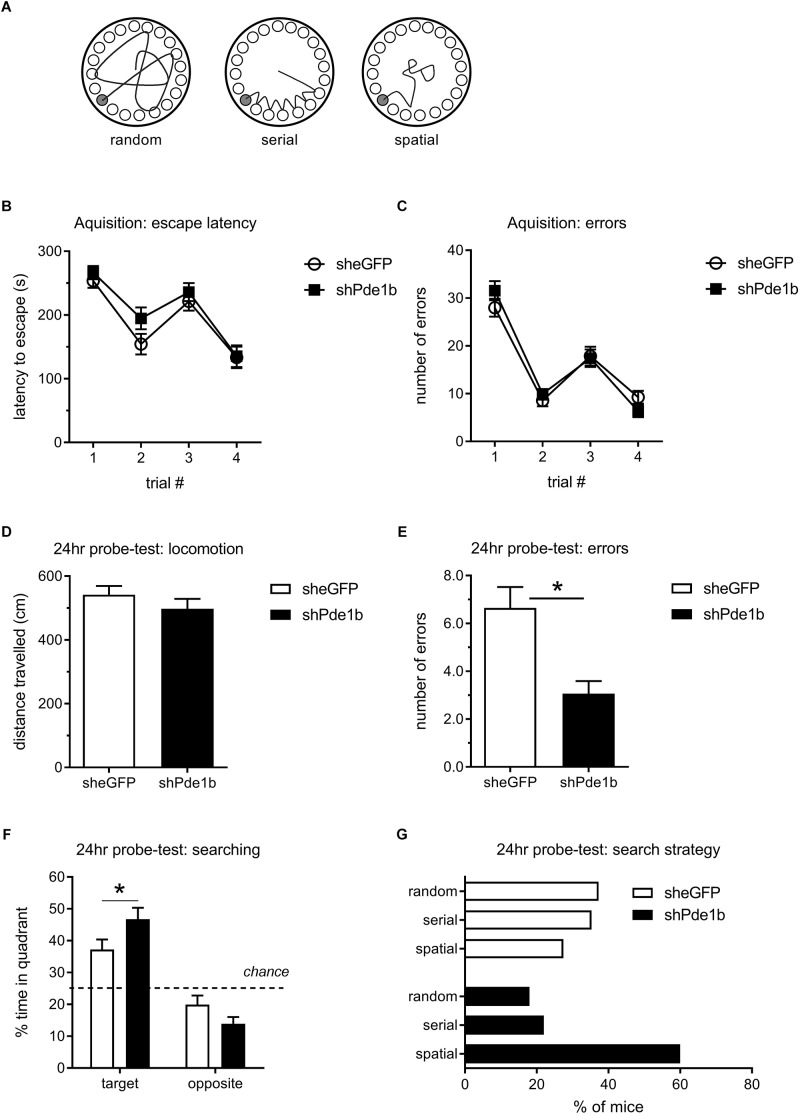
Effects of intra-hippocampal shRNA PDE1B knockdown on spatial memory in the Barnes Maze in mice. **(A)** Search strategies employed in the Barnes Maze test. Mice randomly sample escape holes first, but then quickly revert to serial sampling and finally employ spatial search strategies as training progresses. **(B)** Escape latencies during the 4 acquisition trials, two each day separated by a 1 min ITI, did not differ between the treatment groups. **(C)** The number of errors prior to the correct escape did not differ amongst the treatment groups. Both shPde1b (*n* = 50) and shGFP control (*n* = 51) virus treated mice improved within each training day, indicating normal working memory. D-G) A 2 min probe-test was conducted 24 h after the 4th acquisition trial. **(D)** Locomotion during probe-test did not differ between the treatment groups. **(E)** shPde1b treated mice made significantly less errors before approaching the correct (trained) escape hole. **(F)** shPde1b mice spent significantly more time in the quadrant that contained the trained escape hole than shGFP controls. **(G)** Proportion of mice using random, serial, and spatial sampling strategies. A larger proportion of shPde1b treated mice utilized spatial search strategies. Mean ± s.e.m. are shown. Significant differences from vehicle control are indicated by an asterisk (^∗^).

To explore the role of PDE1B in spatial memory, we tested AAV5 treated mice in the Barnes Maze (Figure 2). We designed a protocol that allowed us to determine treatment effects on acquisition and retention of spatial memory by training our mice for 2 days with 2 trials each day, spaced by a 1 min ITI. This was then followed by a probe test 24 h later to assess long-term retention of spatial information. In the Barnes Maze test of spatial memory, mice tend to first randomly explore the maze but then resort to serial sampling of escape locations, and finally employ spatial strategies to navigate to the correct escape when released from the start location at the center of the maze (Figure 2A; Bach et al., 1999). Our mice quickly learned to escape from the maze with shorter latencies and less errors observed on the second trial of each day when compared to the first, but there was no difference in the latency to escape or in the number of errors made prior to escape between treatment groups – indicating normal acquisition and working memory (effect of trial: latency *F*_(3,297)_ = 41.13, errors: *F*_(3,297)_ = 82.99, *p* < 0.0001 for both; effect of gene and interaction: not significant, RM-ANOVA; Figure 2B,C). In the probe-trial, both treatment groups explored the maze and traveled an equal distance across the 2 min trial (Figure 2D). However, Pde1b shRNA treated mice made significantly less errors before the first approach to the correct escape hole indicating enhanced memory (*t*(99) = 3.50, *p* < 0.001; Figure 2E). In addition, knockdown of PDE1B was associated with increased spatial searching, as Pde1b shRNA treated mice spent significantly more time in the quadrant where the escape was during training (target quadrant: *t*(198) = 2.25, *p* < 0.5; opposite quadrant: *t*(198) = 1.42, *p* = 0.16; Figure 2F). A Chi-squared analysis of the distribution of search strategies employed in the probe-trial revealed a significant shift toward spatial in PDE1B knockdown mice – consistent with hippocampal enhancement (shGFP: 14 spatial, 18 serial, 19 random; shPde1b: 30 spatial, 11 serial, 9 random; χ^2^= 11.07, *p* < 0.01; Figure 2G). Thus, shRNA knockdown of PDE1B enhanced long-term retention of memory and improved spatial navigation in the Barnes Maze test.

**FIGURE 3:**
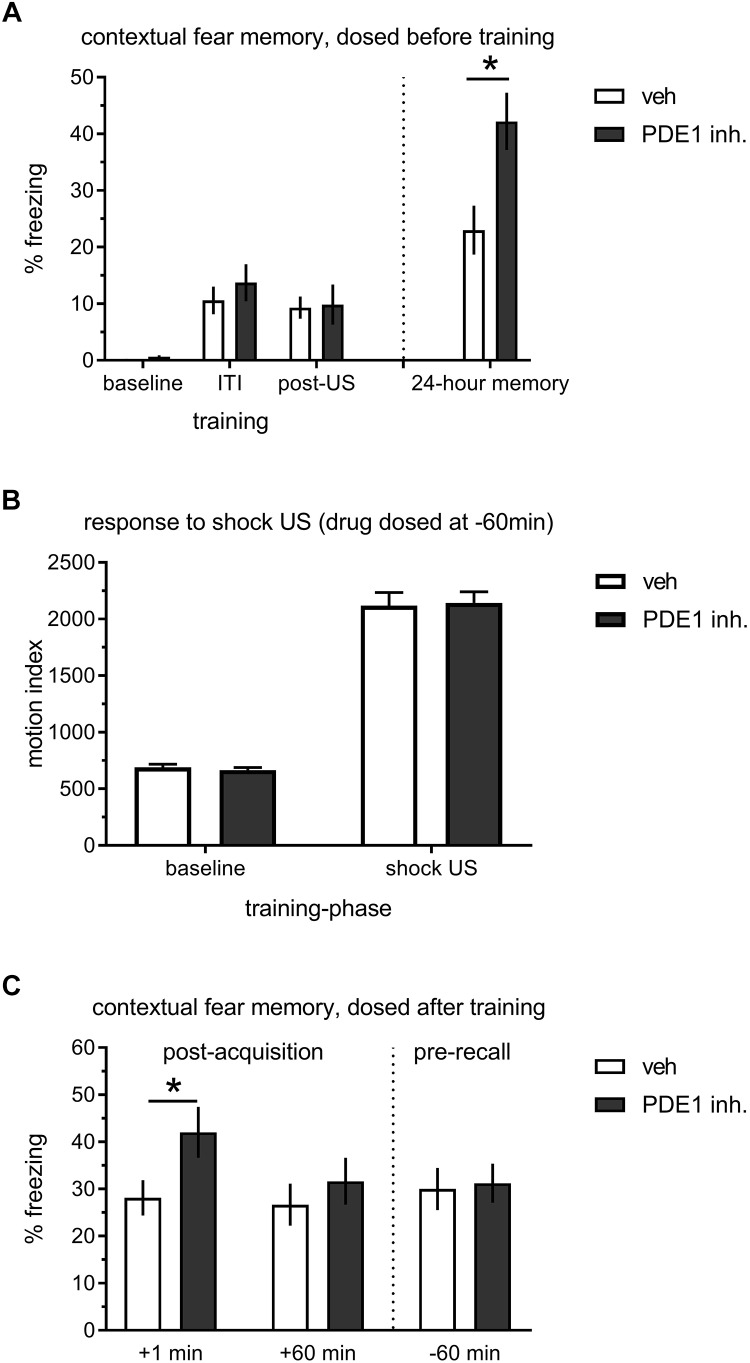
Effect of the PDE1 inhibitor DNS-0056 on acquisition, consolidation, and retrieval of contextual memory in rats. **(A)** 2× trial training 60 min after p.o. administration of DNS-0056 (*n* = 32) or vehicle (*n* = 32), and subsequent 24-h memory test. DNS-0056 did not affect freezing during the acquisition of contextual fear, but significant enhancement was seen in the long-term memory test. **(B)** Response to foot-shock during cFC training, 60 min after a p.o. dose of DNS-0056. The drug did not affect baseline motion or shock US perception. **(C)** Post-trial dosing of DNS-0056 or vehicle. Freezing during the 24-h retention test is shown (*n* = 32 per group/time-point). Drug dosed immediately after training significantly enhanced 24-h retention of contextual fear, but no effect was seen when drug was dosed either 60 min after training, or 60 min before the memory test on day two. Mean ± s.e.m. are shown. Significant differences from vehicle control are indicated by an asterisk (^∗^).

AAV mediated *in vivo* knockdown indicated to us that PDE1B constrains memory formation in the hippocampus, but – because target expression was reduced prior to learning – the effect of PDE1B on encoding, consolidation, and retrieval cannot be differentiated. Thus, we reverted to pharmacological block to more accurately time PDE1B inhibition (Figure 3). We used the novel selective PDE1 inhibitor DNS-0056 – previously demonstrated to facilitate object recognition memory in rats when dosed before training (compound 16-k in Dyck et al. (2017a). Long-Evans rats were given drug, fear conditioned with 2 CS-US pairings, and contextual long-term memory was then tested on the next day. DNS-0056 dosed 1 h prior to training significantly enhanced memory 24 h later (*t*(62) = 2.88, *p* < 0.01), but the drug had no effect on acquisition of contextual fear as evident by similar freezing during the ITI and immediately after the second foot-shock (post-US; Figure 3A). DNS-0056 did not affect baseline locomotion or perception of foot-shock during conditioning, indicating that the drug did not exert obvious sensory-motor effects, consistent with previous observations (Figure 3B; Dyck et al., 2017a). We then proceeded to test the role of PDE1B in consolidation, and dosed DNS-0056 either immediately or 1 h after learning (Figure 3C). When DNS-0056 was administered immediately after contextual conditioning, a significant enhancement of 24 h memory was observed (*t*(62) = 2.12, *p* < 0.05). However, drug given 1 h after training had no effect. In addition, effects on late consolidation were absent when drug was given 3 h after training (data not shown). Finally, we dosed DNS-0056 1 h prior to the memory test on day two. Here again, there was no effect of PDE1B inhibition, indicating that memory recall was not affected. Thus, pharmacological inhibition of PDE1B enhanced consolidation when dosed shortly after learning, but it had no direct effect on memory retrieval.

## Discussion

Our results reveal a role for hippocampal PDE1B as a negative regulator of contextual and spatial memory. Intra-hippocampal knockdown yielded a clear improvement in one-day memory in the contextual fear conditioning task, with no apparent effect on locomotor activity or anxiety related phenotypes. In the Barnes Maze test of spatial memory, memory was enhanced one-day after the end of a two-day training paradigm and this enhancement coincided with a shift toward spatial search strategies in Pde1b shRNA knock-down mice. In addition, pharmacological block of PDE1B at or shortly after training enhanced contextual memory in rats. However, in contrast to inhibitors of PDE2 or PDE4 (Rutten et al., 2007; Peters et al., 2014), PDE1 inhibition did not augment memory when dosed 1–3 h after training. The pharmacological data implicate PDE1 in an early phase of consolidation in the hippocampus, a function that appears distinct from other PDEs.

To our knowledge, the data presented here provide the first clear evidence for a role of PDE1B as a negative regulator of declarative memory. Few suitable compounds exist to interrogate the effects of PDE1 inhibition on memory in animals. Vinpocetine, often cited as a selective PDE1 inhibitor, has only weak activity at this enzyme (PDE1 IC_50_ = 14 μM) (Hagiwara et al., 1984). Moreover, it has considerable off-target activity, binding several other targets with comparable or greater affinity, including benzodiazepine, adrenergic, dopaminergic and adenosine receptors, several ion channels and the IκB kinase (Gulyás et al., 2005; Jeon et al., 2010; Dyck et al., 2017b). While vinpocetine improves spatial memory in rodent models and may increase some measures of cognition in humans, attributing these effects exclusively to PDE1 pharmacology is not possible (DeNoble, 1987; Szatmari and Whitehouse, 2003). A better compound was disclosed in 2016 by Intra-Cellular Therapies Inc. (ITI) (Li et al., 2016; Snyder et al., 2016). This compound (ITI-214) potently inhibits all three PDE1 isoforms and it shows greater than 100-fold selectivity for PDE1B over all other non-PDE1 isoforms with minimal reported off-target activity. In a test of rat object recognition memory, ITI-214 augmented memory at 1 or 3 mg/kg when dosed before training, 3 h after training, or before testing. Additional studies demonstrated a reversal of MK-801 induced working memory deficits in a T-maze spontaneous alternation task, here again at doses of 1–3 mg/kg (Pekcec et al., 2018). In contrast, DNS-0056 only enhanced contextual memory when given either before or immediately after acquisition of contextual fear. While differences in the role of PDE1 in object recognition and contextual memory cannot be entirely excluded, it appears more likely that the discrepancy arises from the much higher exposures of ITI-214 needed for memory enhancement. Total (*C_p_*) and free (*C_p,free_*) plasma levels after 3 mg/kg were estimated at 59 and 0.12 nM, and total brain (*C_b_*) levels of 41 nM based on a B/P of 0.7, respectively (Li et al., 2016; Snyder et al., 2016). The free plasma concentration of ITI-214 is close to the PDE1 Ki values (0.034–0.38 nM) and well below that of other PDEs, but *C_p_* is close to Ki for inhibition of PDE4D (33 nM, also see discussion by Li et al., 2016). PDE4 inhibitors have been demonstrated to facilitate memory at total brain concentrations at or below Ki for PDE4D (Peters et al., 2014; Vanmierlo et al., 2016). Notably, effects of low doses of Rolipram on late consolidation have been reported repeatedly (for example, see Rutten et al., 2006, 2007). Thus, while PDE1 pharmacology certainly contributes to the effects of ITI-214, additive effects of PDE4 inhibition cannot be excluded. Such ‘off-target’ activity is less likely to underlie DNS-0056, effective at total brain concentrations approximately 4× the IC50 to block PDE1B but far below the IC50 for PDE4D (9.3 μM; Bach et al., 1999). In support of this, a related PDE1 inhibitor (example 16-j; Dyck et al., 2017a) also enhanced contextual memory at *C_b_* close to the IC50 for PDE1B (unpublished observation). Despite of the long history of PDE research in both academic and drug discovery settings, few studies have examined the role of the individual PDE isoforms in distinct anatomical regions using molecular genetics. The PDE genes are evolutionary conserved and it is unlikely that their function is redundant. Hippocampal and cortical knockdown of the cAMP specific PDE4D enhances both spatial and contextual memory, and it is associated with increased spine formation, neurogenesis and synaptic plasticity (Rutten et al., 2008; Li et al., 2011; Baumgärtel et al., 2018). Ectopic expression of PDE4A5 impairs memory (Havekes et al., 2016), and induction of the isoform underlies sleep deprivation induced memory impairment (Vecsey et al., 2009). PDE2A, another major target for drug-development, is expressed in a conserved manor amongst mammalian species including in human brain (Stephenson et al., 2009). But homozygous knockout is lethal and pharmacological assessment of its role in cognition have largely relied on a single tool compound – Bay 60-7550 (Schmidt, 2010). PDE1B knockout in mice impairs acquisition in the Morris Water Maze (Reed et al., 2002). The phenotype is opposite to what is shown here, a difference likely explained by neurodevelopmental complications or inverted-U shaped effects. Importantly, however, SKF81297 induced PKA phosphorylation of pSer^845^ GluR1 and pThr^34^ DARPP-32 is augmented in these mice – indicating coupling of PDE1B to D1-R.

Inhibitors of PDE1B may be particularly suited for the treatment of cognitive impairment associated with schizophrenia (CIAS). Current drugs for treating Schizophrenia exert their antipsychotic action via blocking D2 receptors (e.g., Haloperidol) and 5HT-2A receptors (e.g., Risperidone), but they do not affect memory and attentional deficits in patients which are relatively resistant to standard treatment. Pharmacological activation of D1-R has long been considered a possible path toward treating CIAS. Non-clinical studies recently demonstrated the involvement of hippocampal dopaminergic innervations originating in locus coeruleus in spatial object recognition (Kempadoo et al., 2016), as well as in consolidation in a translationally relevant test of ‘everyday’ spatial memory (Takeuchi et al., 2016). This enhancement was blocked by hippocampal D1/5 receptor inhibition, thereby establishing pro-mnemonic effects of D1-R activation. Whether the pro-mnemonic effect of PDE1B inhibition on memory requires D1-R is currently unknown, but knockout mice exhibit increased D1-R signaling (Reed et al., 2002). Abnormal dopamine modulation in the prefrontal cortex of patients with schizophrenia might influence the signal-to-noise ratio in this area and so contribute to the clinical symptoms of schizophrenia (for review, see Rolls et al., 2008). D1-R activation increases NMDA and GABA conductance, and a recent clinical study of intravenous agonist dihydrexidine showed enhanced verbal working memory in schizotypal personality disorder, albeit with side-effects including sedation and cardio-vascular effects (Rosell et al., 2015; Arnsten et al., 2017). To date, no clinical trials testing the effects of PDE1 inhibitors in schizophrenic patients have been reported. However, molecules have advanced to phase 1 safety testing in humans (Dyck et al., 2017b).

In addition to Schizophrenia, PDE1 inhibitors may have therapeutic benefits in mild-cognitive impairment associated with Parkinson’s disease (Parkinson’s MCI), Alzheimer’s disease, and cognitive deficits in major depressive disorder (MDD; Hufgard et al., 2017). However, additional work will be required to determine the mechanistic and behavioral impact of PDE1 inhibition in the context of disease. A better understanding of the mode of activation of PDE1 by Ca^2+^/CaM and the impact of altered Ca^2+^-homeostasis in neurodegenerative disease (Goraya and Cooper, 2005; Gleichmann and Mattson, 2011), of the role of PDE1 in neurogenesis and its contribution to neuropsychiatric conditions and MDD (Ehrman et al., 2006; Li et al., 2011; Kheirbek et al., 2012), and PDE1 modulation of synaptic transmission, plasticity, and spine morphology in disease relevant brain areas (also see, Pekcec et al., 2018) will be paramount. Relevant additional cognitive studies could include evaluating the role of PDE1 in attention and working memory (Pekcec et al., 2018), reward circuits, goal directed learning, as well as the impact on the filtering of relevant and interfering information during memory performance (pattern separation) – which is impaired in MDD, schizophrenia, and aMCI (Leal and Yassa, 2018).

We have shown here that PDE1B is a key regulator of long-term memory in the hippocampus. PDE1B contributes to an early phase of memory consolidation with possibly additional roles in attention and working memory. Further investigation of this novel target for cognitive enhancement, and translational research to determine its utility for treating patients suffering from cognitive impairment, is clearly warranted.

## Significance Statement

There is great need for memory augmenting therapies for patients suffering from cognitive impairment. Drug discovery has been focused on the development of phosphodiesterase inhibitors with memory enhancing properties. PDE4 inhibitors are well known to enhance memory, but the target is associated with dose limiting complications such as emesis and gastrointestinal effects. More recently, medicinal chemistry efforts have led to the discovery of selective inhibitors of other PDEs, including PDE1. However, the role of the PDE1 isoforms in memory is poorly understood as knockout mice suffer from memory deficits, and pharmacological proof may be confounded by compound selectivity. Our work clarifies the role of the PDE1B isoforms in hippocampus, and we show that PDE1 regulates early consolidation using a selective inhibitor. This work establishes the enzyme as a target for enhancement of hippocampal memory, and the findings are of relevance for the development of drugs to treat memory deficits in schizophrenia, depression, and neurodegenerative conditions.

## Author Contributions

MP designed the research and wrote the manuscript with input from BD and KB. RS designed the research and edited the manuscript. SX, KB, SM, RB, and GA conducted the research.

## Conflict of Interest Statement

The authors declare that the research was conducted in the absence of any commercial or financial relationships that could be construed as a potential conflict of interest.
